# Modeling the Impact of Soil and Water Conservation on Surface and Ground Water Based on the SCS and Visual Modflow

**DOI:** 10.1371/journal.pone.0079103

**Published:** 2013-11-11

**Authors:** Hong Wang, Jian-en Gao, Shao-long Zhang, Meng-jie Zhang, Xing-hua Li

**Affiliations:** 1 Institute of Soil and Water Conservation, Chinese Academy of Sciences and Ministry of Water Resources, Yangling, Shaanxi Province, China; 2 Institute of Soil and Water Conservation, Northwest A&F University, Yangling, Shaanxi Province, China; 3 University of Chinese Academy of Sciences, Beijing, China; 4 College of Water Resources and Architectural Engineering, Northwest A&F University, Yangling, Shaanxi Province, China; 5 College of Natural Resources and Environment, Northwest A&F University, Yangling, Shaanxi Province, China; Catalan Institute for Water Research (ICRA), Spain

## Abstract

Soil and water conservation measures can impact hydrological cycle, but quantitative analysis of this impact is still difficult in a watershed scale. To assess the effect quantitatively, a three-dimensional finite-difference groundwater flow model (MODFLOW) with a surface runoff model–the Soil Conservation Service (SCS) were calibrated and applied based on the artificial rainfall experiments. Then, three soil and water conservation scenarios were simulated on the sand-box model to assess the effect of bare slope changing to grass land and straw mulching on water volume, hydraulic head, runoff process of groundwater and surface water. Under the 120 mm rainfall, 60 mm/h rainfall intensity, 5 m^2^ area, 3° slope conditions, the comparative results indicated that the trend was decrease in surface runoff and increase in subsurface runoff coincided with the land-use converted from bare slope to grass land and straw mulching. The simulated mean surface runoff modulus was 3.64×10^−2^ m^3^/m^2^/h in the bare slope scenario, while the observed values were 1.54×10^−2^ m^3^/m^2^/h and 0.12×10^−2^ m^3^/m^2^/h in the lawn and straw mulching scenarios respectively. Compared to the bare slope, the benefits of surface water reduction were 57.8% and 92.4% correspondingly. At the end of simulation period (T = 396 min), the simulated mean groundwater runoff modulus was 2.82×10^−2^ m^3^/m^2^/h in the bare slope scenario, while the observed volumes were 3.46×10^−2^ m^3^/m^2^/h and 4.91×10^−2^ m^3^/m^2^/h in the lawn and straw mulching scenarios respectively. So the benefits of groundwater increase were 22.7% and 60.4% correspondingly. It was concluded that the soil and water conservation played an important role in weakening the surface runoff and strengthening the underground runoff. Meanwhile the quantitative analysis using a modeling approach could provide a thought for the study in a watershed scale to help decision-makers manage water resources.

## Introduction

In recent years, with more and more officials and professionals paying attention to the problems such as soil erosion and water loss, the contradiction between water supply and demand, whether soil and water conservation measures on upper regions would have effect on the amount of water resources of the lower reaches is a scientific issue to be discussed and probed into urgently [1]. For many years, the work of soil and water conservation as same as the Yellow River management, not only obtains great achievement, but also brings some problems [2]. The biggest achievement was no more than soil erosion control in the watershed, and thereby reducing the amount of sediment flowed into the river. Took the Yellow River basin for example, changes in land use and vegetation in the Loess Plateau had a decisive impact on sediment transport of Yellow River [3], even about 60% sediment yield since 10 ka BP occurred during the last 1040 years of the period of estrepement in Loess Plateau. The sediment monitoring at Sanmenxia hydrological station of Yellow River showed the annual average sediment discharge was 1040 million tons during 1950–2010, 589 million tons during 1987–2010, while it was only 198 and 351 million tons in 2009 and 2010 separately [4].

The reduction in the quantity of the sediment discharge by soil and water conservation is no doubt pleasing, but it also brings a new eco-environmental problem for its surface water reduction function. Some studies found that land use/land cover (LULC) change could alter hydrological cycles by affecting ecosystem evaportranspiration, soil infiltration capacity, surface and subsurface flow regimes [5]–[8]. Just as the study of the relation between control in Loess Plateau and no-flow in the Yellow River showed that the influence of water reduction on no-flow in the lower Yellow River for the comprehensive control changed the condition underlying surface and influenced the water cycle was nonnegligible [9]. Results of soil and water conservation method showed that the mean flood reduced 0.5456 billion tons in He-Long section of middle Yellow River, Jinghe, Beiluohe and Weihe watershed from 1970 to 1996 [10]. The average runoff reduction benefits of soil and water conservation measures for Chabagou, Dalihe and Wudinghe basins in the 1970s were 14.47%, 20.22% and 20.78% respectively [11]. Jain and Mishra et al [12] evaluated the suitability to particular land use, soil type and combination a quantitative evaluation of the existing Soil Conservation Service Curve Number (SCS-CN) model using a large set of rainfall-runoff data from small to large watersheds of the U.S.A. Shi and Yi et al [13] evaluated the effect of land use/cover change on surface runoff by SCS model in Shenzhen region, China. Guo and Wang et al [14] calculated the volume of surface runoff during 5 rainfalls on 5 different kinds of land use types in sloping runoff plots by SCS model.

Soil and water conservation could reduce surface runoff and it is bound to affect groundwater recharge, which is the entry into the saturated zone of water made available at the table surface, together with the associated flow away from the water table within the saturated zone [15]. But less direct study has previously been undertaken to assess this effect quantitatively. It had been shown that groundwater recharge was closely related to land-use types very much. The impact of land-use on distributed groundwater recharge and discharge in the western Jilin, China, using MODFLOW, WetSpass, the Seepage packages, and ArcGIS showed that forest vegetation had the highest recharge, followed by agricultural farmlands and the recharge generally decreased when vegetated forests deteriorate to be other landforms (bush, grassland or bare-land) [16]. A study by Cho and Barone using MODFLOW indicated that subsurface flow regimes could be negatively affected by urbanization due to increased withdrawal and reduced recharge [17]. A number of studies showed the effects of changes in forest cover on groundwater recharge [18]–[22]. These studies showed a range in the reduction of groundwater recharge beneath trees, from 15% to 90%, compared to that under grass [18]. Thus the impacts of land-use on the atmospheric components of the hydrologic cycle (regional and global) are increasingly being recognized, though those on the subsurface components of the hydrologic cycle, particularly groundwater recharge are not equally known [23].

Therefore special attention should be given to the effect of soil and water conservation on the hydrologic cycle, especially recharge and discharge. Both monitoring and modeling approaches are used for conjunctive investigation of surface water and groundwater. The monitoring approach is expensive and time demanding yet measuring actual changes in stream and groundwater levels over time may lead to more direct estimates of the impact of land development on both surface and subsurface flows [17]. Through the model coupling approach, interaction between surface water and groundwater has become a trend [24], [25]. Linkage between MODFLOW and existing surface models such as the SCS method to consider surface water and groundwater interactions could validate the outputs of the model.

The overall goal of this study is to assess the impact of soil and water conservation on the surface and subsurface flow by mathematical models combined with artificial rainfall simulation experiments and gives a quantitative analysis thought simultaneously.

## Materials and Methods

### SCS Curve Number Method

The runoff equation of the U.S. Soil Conservation Service, commonly called the curve number method, for estimating runoff from rainfall, came into common use in the mid - 1950s [26]. Although the SCS method for runoff estimation has changed little since the 1960s, its popularity has been maintained over the years [27]. The SCS-CN equation, in the typical form [28], is given as:
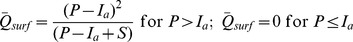
(1)


Where 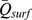 is the surface runoff (mm), in this study it is the average runoff depth per hour; P is the precipitation (mm), in this study it is the precipitation per hour; S is the amount of water storage available in the soil profile or the maximum storage (mm), and I_a_ is the initial abstraction (mm). To reduce the numbers of variables, the empirical relationship I_a_ = O.2S was adopted, which then gives the most familiar form of the runoff equation [29]:
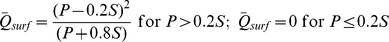
(2)


Because the range of the S value is too large to obtain a suitable value, the dimensionless parameter, curve number (CN), was introduced into this formula. The parameter is related to CN by the relationship:
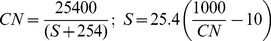
(3)


The CN is a comprehensive parameter which related to initial soil moisture, slope, vegetation, soil type and land use status etc [30]. It appeared that the curve numbers were used as a proxy for the retention parameter S in order to scale the curves to a convenient range between zero and 100 [29].

Meanwhile, the runoff amount could be calculated combined the rainfall duration by the relationship:
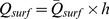
(4)


Where 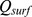 is the total runoff of rainfall (120 mm) (mm); h is the rainfall duration (h).

Relative error (RE) was selected as the statistical evaluation index of SCS and it was defined by the equation below:
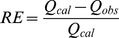
(5)


Where Q_cal_ was the calculated runoff by SCS model (mm); Q_obs_ was the observed runoff obtained from the rainfall experiment (mm).

### Visual Modflow Model

MODFLOW (Modular Three-dimensional Finite-difference Ground-water Flow Model)) is a modular finite-difference ground-water flow model published by the U.S. Geological Survey, which can simulate ground-water flow in a three-dimensional heterogeneous and anisotropic medium. Using the finite-difference method, the domain in which flow is to be simulated is divided into a rectilinear mesh of rows, columns, and layers [31]–[33]. Visual MODFLOW is also a three-dimensional groundwater flow and contaminant transport modeling application that integrates MODFLOW, MODPATH, MT3DMS, WinPEST, Zone Budget, and so on. Applications include well head capture zone delineation, pumping well optimization, aquifer storage and recovery, groundwater remediation design, simulating natural attenuation, and saltwater intrusion [32].

The three-dimensional movement of ground water of constant density through porous earth material may be described by the partial-differential equation [31]:
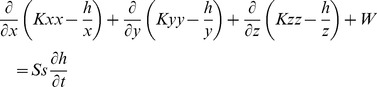
(6)where Kxx, Kyy, and Kzz are values of hydraulic conductivity along the x, y, and z coordinate axes, which are assumed to be parallel to the major axes of hydraulic conductivity (m/s); h is the potentiometric head (m); W is a volumetric flux per unit volume representing sources and/or sinks of water, with W<0 for flow out of the ground water system, and W>0 for flow into the system; Ss is the specific storage of the porous material (1/m); and t is time (min).

The calibration statistics can be reported in the result plots and the statistical evaluation indexes, including Calibration Residual (CM), Residual Mean (RM), Absolute Residual Mean (ARM), Standard Error of the Estimate (SEE), Root Mean Squared (RMS), Normalized Root Mean Squared (NRMS) and Correlation Coefficient (Cor) [32]. Based on the principle of the modeling and simulated rainfall experiments, a transient three-dimensional groundwater flow model was built using Visual MODFLOW 4.1. The model domain was divided into a 50×265 array of 4×10^−4^ square meter cells uniformly. The model consisted of three individual layers with gradient of 3° and the thicknesses of layers were respectively 0.5, 1 and 98.5 cm from top to bottom. The transient flow simulation was selected as the flow type and the time unit for all parameters was minute. The edge of the model domain was modeled as a no-flow boundary. Actual quantities of groundwater abstraction from drain pipe were treated as flux boundary condition in form of pumping well. Groundwater recharge from rainfall was modeled as recharge package in MODFLOW. The initial ground water level was 0.39 m high and paralleled to the bottom of the model. The initial value and the range of hydraulic conductivity and storage coefficient values at different layers were assigned to each active grid cell by interpolation of discrete property data derived from water releasing test analysis and regional geology data.

### Simulated Rainfall Experiments

#### Experimental conditions and equipment

**Figure 1:**
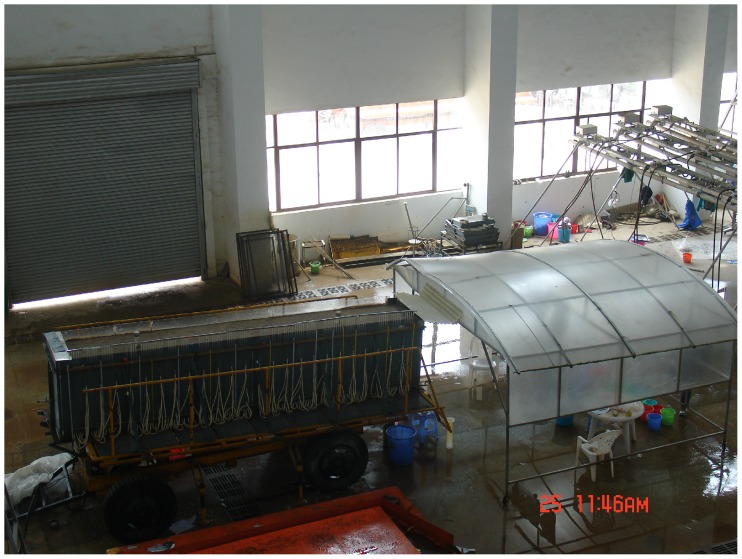
The experimental flume.

The simulated rainfall experiments were carried out in the Rainfall Simulation Hall of the State Key Laboratory of Soil Erosion and Dryland Farming on the Loess Plateau during the period of June to October of the year 2012. The simulated rainfall system, with automatic simulation device of under sprinkler, could ensure the kinetic energy of simulated precipitation close to the natural rainfall for the mean fall-height of 18 meters. And the calibration tests showed that rainfall uniformity was greater than 85%. The experiments were conducted in the sand-box model [34], [35] (figure 1), 5.3 m×1 m×1 m at the Rainfall Simulation Hall. The slope of the model could be adjusted manually from 0° to 35°. There were two water tanks, 0.15 m×1 m×1 m in front and back of the model, for the regulation of the groundwater level. Above the front side of the water tank there was one surface water groove and there was one drainage pipe of groundwater in the 0.39 m high at the front side of the water tank.And one hundred and twenty 0.2 m×0.2 m sets of piezometric tubes of level observation were installed in left side of the sand-box model. And two neutron probe access tubes down to a depth of 0.9 m in experimental flume for soil moisture control.

The experimental flume was fixed at an angle of 3° in this study. Three water and soil conservation measures (bare slope, straw mulching and grass land) were considered and the soil surface condition should be roughen to keep the beginning condition consistent every time before experiment. The precipitation for a control to be equal was 120 mm. Six gradient rainfall intensities (45∼120 mm/h) with uniform rainfall conditions were simulated for 160 to 60 min correspondingly. The initial ground water level was parallel to the bed of the flume and the distance between two lines was 0.39 m.

#### Experimental materials and monitoring methods

**Figure 2:**
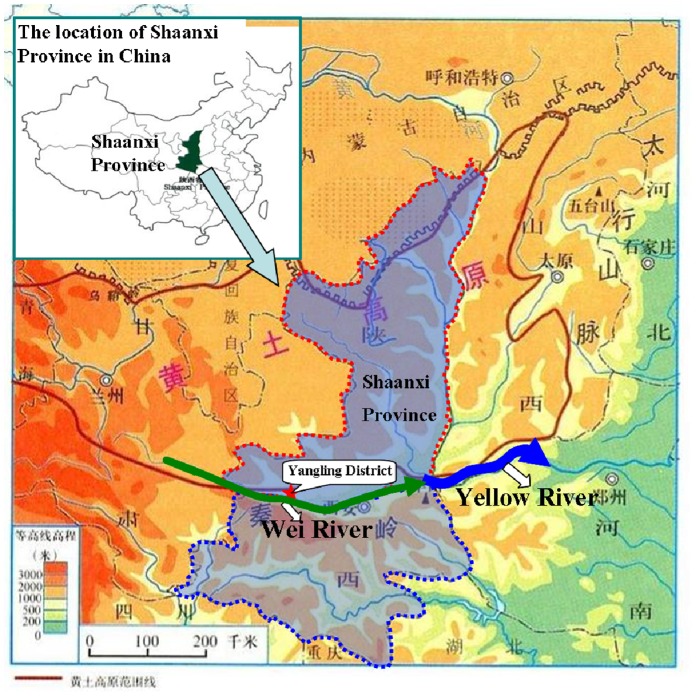
The schematic map of Wei River and Yangling District location.

The test materials included riversand and Lou soil. The riversand samples were dug from the middle and lower reaches of the Wei River bank in Yangling District and the Lou soil was also collected from Yangling District, Shaanxi Province, China. Figure 2 is the schematic map of Wei River and Yangling District location. The mechanical composition of soil particles was shown in table 1. The samples were air-dried for about ten days and sieved through a series of corresponding magnitude sieves. Then the test materials were packed into the flume layer by layer. The experimental flume consists of three individual layers and the thickness was respectively 0.5, 1 and 98.5 cm from top to bottom. The medium sand (0.25 mm ∼ 0.5 mm) was paved in the third 98.5 cm deep layer and the soil bulk density was 1.4∼1.5 g/cm^3^. In the second layer the riversand particle size was less than 0.25 mm and the average soil bulk density was about 1.6 g/cm^3^. On the top layer, the soil used was the composite sandy loam, composed of riversand (<0.25 mm) and Lou soil, the weight ratio of this composite soil was about 2∶5 and the average soil bulk density was also about 1.6 g/cm^3^. The setting of water and soil conservation measures as follows: for the straw mulching, the quantity of arid straw mulching was designed as 0.4 kg/m^2^ uniformly each experiment with coverage of 85∼90%; for the grassland, the grass was grew on the soil in the flume and its species was ophiopogon japonicus with coverage of 65%∼70% and the planting structure was, 10 cm row spacing×5 cm plant spacing×8.2 cm average plant height.

**Table 1:** Mechanical composition of experimental materials (%).

Types	1∼0.5	0.5∼0.25	0.25∼0.05	0.05∼0.01	0.01∼0.005	0.005∼0.001	<0.001
Lou soil	0.07	0.65	5.86	49.04	12.18	13.72	18.48
Mixed soil	1.83	24.34	30.91	25.29	4.82	5.53	7.27

The main monitoring items measured during the experiments were: the surface runoff amount, the surface runoff in the process, underground runoff and groundwater level etc. Surface runoff was collected by collecting buckets at the beginning of runoff yield. The surface runoff in the process was collected for 30 seconds at 5-min intervals during experiments and the runoff amount was collected during the whole process. After groundwater level adjustment, recorded the initial level first and the dynamic changes every 10 minutes with the changes begin for about 4 hours. Meanwhile, underground runoff was collected by measuring cylinder per 2000 ml uninterruptedly for about 8 hours. Then according to the condition of the underground runoff, the monitoring interval was lengthened gradually until the water level dropped to the initial control water level. Soil moisture (neutron probe method) measurements were taken immediately before and after precipitation to ensure replicability of the initial soil surface conditions. And water temperature was also recorded during each simulation.

### Ethics Statement

No specific permissions were required for these sampling activities because the location is not privately-owned or protected in any way and the field activities did not involve endangered or protected species.

### The Benefits of Water Quantity Change for Soil and Water Conservation Measures

The quantity of surface water and groundwater could be changed by adoption of conservation management practices. Quantitative analysis of this change could be computed by the relationship:
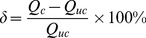
(7)


Where 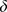 is the benefit of water change contributed by soil and water conservation measures, %; 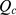 is the runoff modulus in the scenario controlled by soil and water conservation; 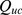 is the runoff modulus in the bare slope scenario.

## Results and Discussions

### Model Calibration and Validation

Model calibration is the process whereby selected model input parameters are adjusted within reasonable limits to produce simulation results that best match the known or measured values [32]. It is the most critical process in building a model, because the quality of the calibration and validation inevitably determines the reliability of any conclusions and recommendations made using the simulation results.

### Calibration and Validation of SCS Model

Where concurrent rainfall and runoff data are available, an ‘optimal’ curve number can be found by calibrating the curve number to the data [29]. Based on the rainfall runoff data obtained from simulated rainfall experiment for the rainfall intensity 75 mm/h of bare slope scenario, the SCS model was calibrated. Observed 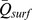 and P were 50.01 mm and 75 mm respectively. According to eq. (2) and (3), S and CN values were calculated successively. The calibrated CN value was 90.33. Then based on precipitation data from rainfall intensity 45, 90, 105, 120 mm/h of bare slope scenarios respectively, the SCS model were verified by comparing the estimated runoff with in situ measured data.

Based on the statistical analysis of model results, the relative error (RE, eq. 5) in the estimated runoff ranged from 0.40 to 3.41% (table 2) during the verification period respectively. The calibration and verification statistics showed the model did a relatively good job for surface water predicting. Hence, the SCS model was available for the predicting in this study. So surface runoff of bare slope scenario could be simulated by the verified SCS model and the calculated result was compared with the corresponding observed runoff of lawn and straw mulching experiments to study the impact of soil and water conservation on surface water.

**Table 2:** Calibrated parameter value and the relative error (RE) (%).

Period	Calibrationperiod	Verification period			
Rainfall Intensity(mm/h)	75	45	90	105	120
RE (%)	0.015	3.41	0.40	2.20	1.77

### Calibration and Validation of Visual MODFLOW Model

The main parameters of the model were: hydraulic conductivity (Kxx, Kyy, and Kzz), storage (specific storage (Ss), specific yield (Sy)). The initial values and the range of these parameters at different layers were provided by the water releasing test analysis combined with the regional geology data. These data were imported into the constructed MODFLOW model as the initial parameters and adjusted during the process of model calibration. Then based on the simulated rainfall experiment of the bare slope, which intensity was also 75 mm/h and rainfall was 120 mm, a uncertainty analysis was carried out after calibration to quantify the uncertainty in the calibrated model caused by uncertainty in the estimates of aquifer parameters, recharge boundary conditions, initial head conditions and river-aquifer interactions [18]. The uncertainty analysis showed that horizontal conductivity in the third layer (Kx3), specific yield (Sy) were the most sensitive parameters in this model. The main parameters calibrated were shown in table 3. Then to verify the model used another simulated rainfall experiment data of the bare slope, which intensity was 45 mm/h and rainfall was also 120 mm.

**Table 3:** The main calibrated parameters of the MODFLOW model.

Parameters	Kx1(m/s)	Kz1(m/s)	Kx2(m/s)	Kz2(m/s)	Kx3(m/s)	Kz3(m/s)	Sy	Ss
Minimum	1.16×10^−8^	1.16×10^−9^	5.79×10^−7^	5.79×10^−8^	5.79×10^−5^	5.79×10^−6^	0.05	1×10^−5^
Maximum	5.79×10^−6^	5.79×10^−6^	5.79×10^−5^	5.79×10^−5^	5.21×10^−4^	5.21×10^−4^	0.5	1×10^−3^
Initial Value	2.90×10^−6^	2.90×10^−7^	3.18×10^−5^	3.18×10^−6^	2.2×10^−4^	2.2×10^−5^	0.2	1×10^−4^
Calibrated Value	1.12×10^−7^	2.52×10^−9^	1.50×10^−6^	7.47×10^−7^	3.1×10^−4^	7.60×10^−5^	0.36	1.385×10^−4^

**Figure 3:**
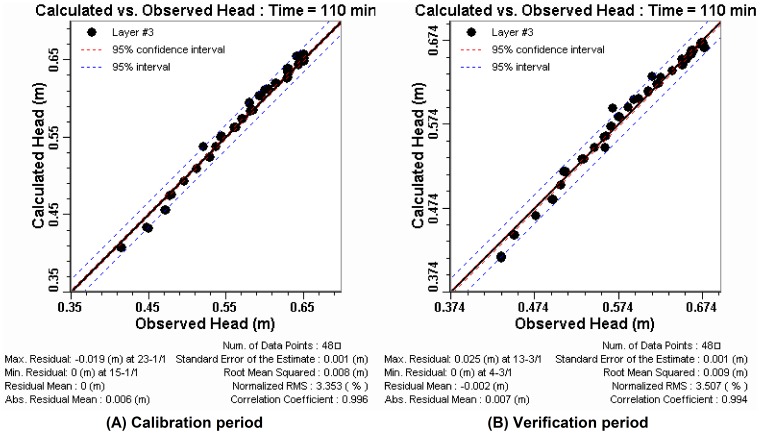
The scatter graph of calculated vs. observed values.

The scatter graph of calculated vs. observed values was the default calibration graph. This graph represents a snap-shot in time of the comparison between the values calculated by the model (Y-axis), and the values observed or measured in the field (X-axis) [32]. For example, the scatter graphs at t = 110 min of the calibration period and the verification period (figure 3) showed that most of the data points intersect the 45 degree line on the graph where X = Y. These represented an ideal calibration that simulated hydraulic heads were consistent with the observed heads. The calibration statistics were reported in the footer of the calibration plots window when the calculated vs. observed scatter graph were displayed [32]. Based on these statistical analysis of model results, error indexes including the residual mean (RM), the absolute residual mean (ARM), the standard error of the estimate (SEE), the root mean squared error (RMS), the normalized root mean squared (NRMS) and the correlation coefficient (Cor) for groundwater levels at the location of 48 observation boreholes during the calibration period and the verification period respectively were shown in table 4.

**Table 4:** Error indexes of calculated vs. observed values.

EvaluationIndexes	RM/m	ARM/m	SEE/m	RMS/m	NRMS/%	Cor
Calibration	0.0002	0.00727	0.0012	0.0097	4.146	0.996
Verification	0.000375	0.00843	0.00119	0.00981	4.259	0.994

Based upon comparisons the scatter graphs and the calibration statistics between the observed and simulated hydraulic head, we could see the groundwater levels simulated by the calibrated groundwater flow model were generally consistent with the physical system represented.

### The Impact of Soil and Water Conservation on Surface and Ground Water

Then the calibrated and verified SCS and MODFLOW models were used to simulate rainfall intensity 60 mm/h of bare slope scenario to research the hydrologic impact of soil and water conservation. First, surface runoff amount was simulated by the SCS model and was compared with the corresponding results of lawn and straw mulching experiments to study the impact for the surface water. Second, the calibrated groundwater transient flow model with the input levels obtained from corresponding lawn and straw mulching experiments was used to assess the impact of land change on groundwater.

### The Impact of Soil and Water Conservation on Surface Water

The surface runoff modulus amount calculated by SCS model was 0.364 m^3^ in bare slope scenario, while the surface runoff observed from the rainfall experiments were 0.154 m^3^ and 0.012 m^3^ in the lawn and straw mulching scenarios respectively. Compared to the bare slope scenario, the benefits of surface water reduction by these two measures were 57.8% and 92.4% correspondingly. These results indicated that the soil and water conservation played an important role in the benefits of water reduction. These were consistent with the field observation results of the small watersheds. For example, by comparing the 12 groups (24) of controlled and uncontrolled small watersheds (table 5) in Shaanxi Province, China [36], we could see the benefits of surface water reduction by the comprehensive control of soil and water conservation varied from 14.71% to 88.19%, while the corresponding average value was 44.09%.

**Table 5:** The benefits of water reduction in field controlled and uncontrolled small watersheds %.

Water System	Watershed Name	Area (km^2^)	Condition	Representative Type Area	Year	Benefits δ(%)
Kuye River	Mengjiagou	2.03	Controlled	Earth-rocky Mountainous Area	1959∼1961	42.64
	Yangyagou	1.88	Uncontrolled			
Yuxi River	Qingcaogou	0.373	Controlled	Half sandstorm area	1959∼1960	88.19
	Wangjiagou	0.434	Uncontrolled			
	Tiaogou	0.7677	Controlled		1959∼1961	16.51
	Lijiagou	0.693	Uncontrolled			
Wuding River	Jiuyuangou	70.1	Controlled	Loess Hilly and Gully Region	1959∼1969	16.88
	Peijiamaogou	41.2	Uncontrolled			
	Xiangtagou	0.454	Controlled		1958∼1961	23.70
	Tuanyuangou	0.491	Uncontrolled			
	Wangmaozhuanggou	5.967	Controlled		1962∼1963	43.70
	Lijiazhaigou	5.45	Uncontrolled			
Yanhe River	Dabiangou	3.7	Controlled		1963∼1967	39.63
	Xiaobiangou	3.925	Uncontrolled			
Luohe River	Sigou	4.37	Controlled	gully region of loess plateau	1959∼1961	57.98
	Nangou	5.11	Uncontrolled			
Juhe River	Guanzhuanggou	3.39	Controlled		1959∼1961	57.98
	Yuanguzhuanggou	2.82	Uncontrolled			
Luohe River	Xingshugou	0.522	Controlled	Loess Hilly and Gully Region	1958∼1961	83.62
	Beilougou	0.334	Uncontrolled			
Jinghe River	Fengyugou	1.176	Controlled		1958∼1960	14.71
	Wangjiagou	0.874	Uncontrolled			
Chanhe River	Yaojiagou	7.815	Controlled	Terrace region of loess plateau	1960∼1961	64.99
	Dicungou	4.4	Uncontrolled			
Average						44.09

**Figure 4:**
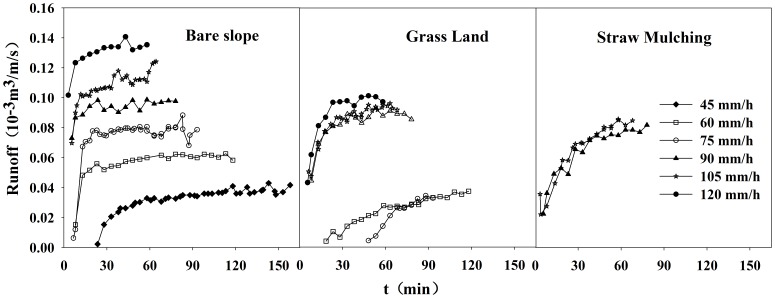
The time-series graphs of the surface runoff under three simulation scenarios.

Not only the amount of surface runoff, but also the runoff process was important for studying the hydrologic impact of soil and water conservation. In this research, the SCS model could reliably estimate runoff amount, but it could not predict runoff process. So the influence for the process of surface runoff was analyzed using simulated rainfall experiments. The time-series graphs (figure 4) were used to evaluate and compare temporal trends in the runoff under three simulation scenarios. The process of runoff would appear to increase first and then stabilize gradually with the passage of rainfall time under three simulation scenarios. This result was consistent with the research that rainfall infiltration rate was decreased first and then stabilize gradually with the passage of rainfall time [37].

**Figure 5:**
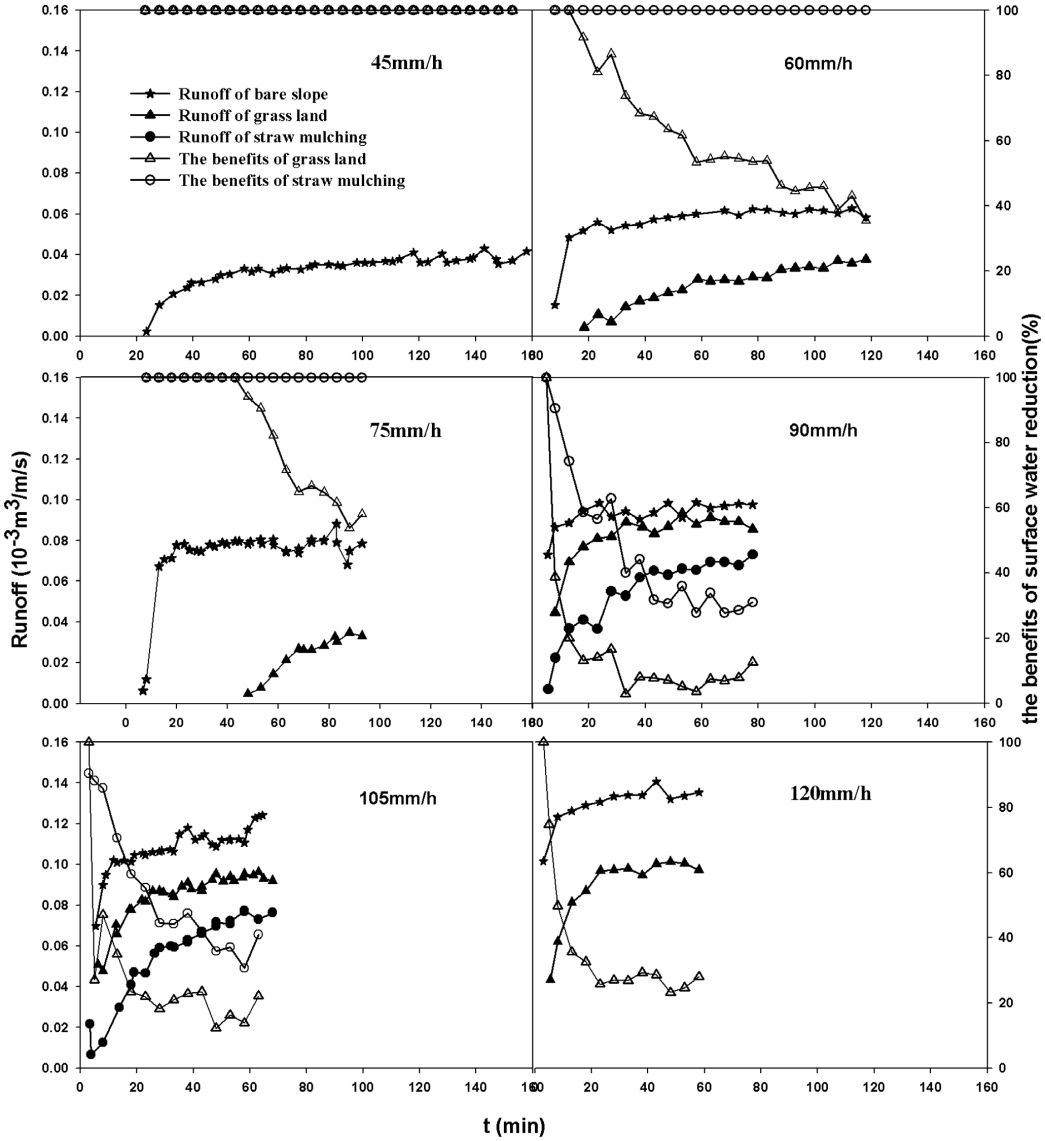
The time-series graphs of the surface runoff and the benefits of surface water reduction.

Compared with the bare slope scenario, the reduced range of runoff was different in the lawn scenario and in the straw mulching scenario. When rainfall intensity increased from 75 mm/h to 90 mm/h, the latter average runoff was 7.3 times larger than that of the former in the lawn scenario and it started runoff in the straw mulch scenario. During the whole runoff process of the experiments, the benefits of surface water reduction by the grassland varied from 24.3% to 100% and its average value was 58.9%, while the range was 55.3% to 100% and the average value was 84.2% in the straw mulching scenario (figure 5).

### The Impact of Soil and Water Conservation on Ground Water

**Figure 6:**
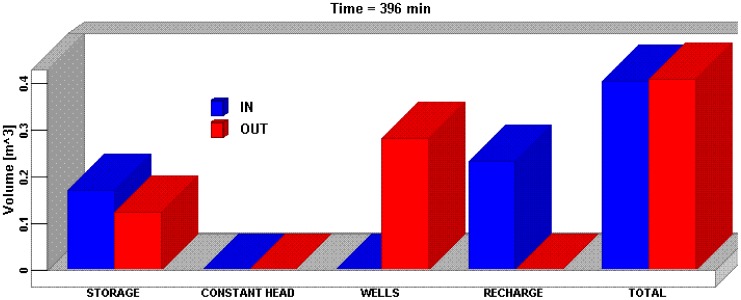
The flow mass balance graph.

As mentioned above, the calibrated and verified MODFLOW model was then applied to simulating the impact of soil and water conservation scenarios on groundwater recharge and level. The mass balance which is one of the key indicators of a successful simulation [32] was analyzed first. The flow mass balance graph (figure 6) showed the volume of water entering and leaving the system through the flow boundary conditions, and from aquifer storage at the end of simulation period. The total volume flow into the entire system was 0.4059 m^3^ which included 0.1719 m^3^ storage and 0.2345 m^3^ recharge. The total volume flow out-of the model was 0.4070 m^3^ which consisted of 0.1252 m^3^ storage and 0.2818 m^3^ well. The mass balance error for the simulation inflow and outflow was 1.47% <2%. The results of the simulation may generally be considered acceptable, provided the model was also calibrated [32].

**Figure 7:**
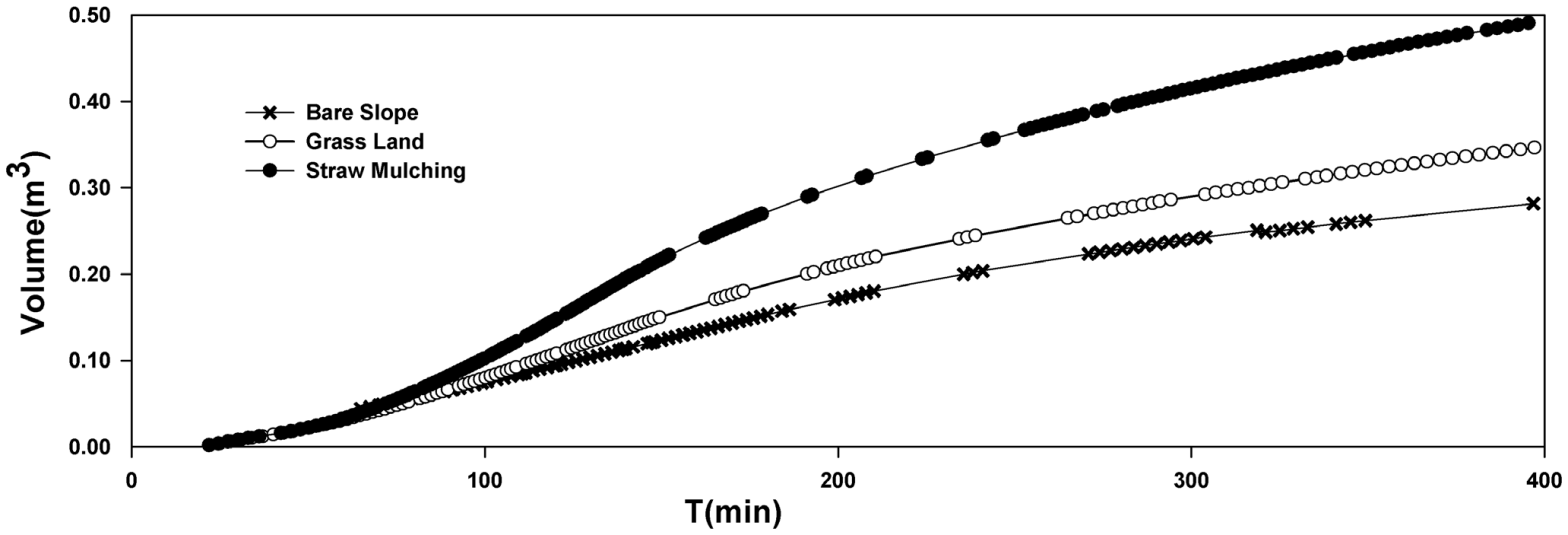
The time-series graphs of accumulated underground water volume under three simulation scenarios.

Figure 7 compared the changes in accumulated underground runoff with scenarios converted from bare slope to grass land and straw mulching. As a result, the cumulative curve of straw mulching was the highest, the grassland took second place, and the bare slope was the lowest. Hence, there was a significant increase for straw mulching, contrasting to the changes for grassland. Then the impact of groundwater cumulant was analyzed quantitatively. At the end of simulation period, the accumulated underground runoff was 0.282 m^3^ in the bare slope scenario, 0.346 m^3^ in the lawn scenario, and 0.491 m^3^ in the straw mulching scenario. Compared to the bare slope scenario, the amount and the benefit of groundwater recharge increased by 0.064 m^3^ and 22.7% respectively in the lawn scenario. Accordingly, they were 0.209 m^3^ and 60.4% in the straw mulching scenario. It was explicit that groundwater recharge very strongly depended upon land-use type [16].

**Figure 8:**
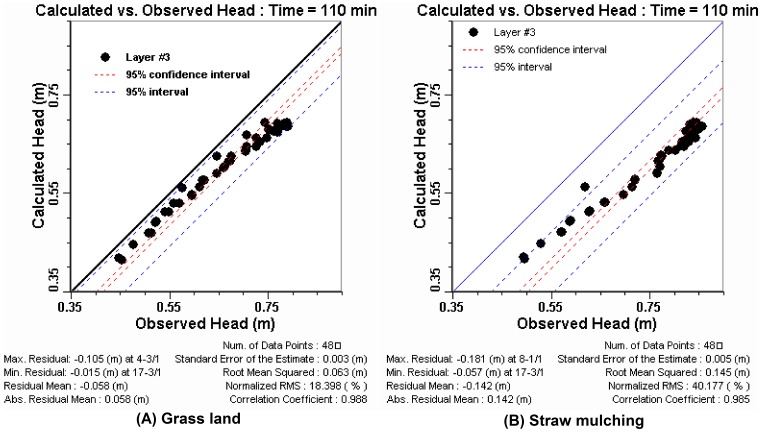
The scatter graph of calculated vs. observed values.

The groundwater levels responded to the changes in groundwater recharge described as follows. Figure 8 compared the influence of soil and water conservation scenarios on groundwater levels at t = 110 min. Figure 8 (A) was the scatter plots of calculated levels of bare slope scenario vs. observed heads of grass land scenario. And figure 8 (B) was the graph of calculated levels of bare slope scenario vs. observed values of straw mulching scenario. As shown in the figures, the data points were under the X = Y line, so the calculated values were less than the observed values, especially in figure 8 (B). These indicated that groundwater levels increased with land-use converted from bare slope to grassland and even more with land-use converted to straw mulching. To quantify the impact on groundwater heads, the changes between the calculated heads of bare slope scenario and observed levels of grass land and straw mulching scenarios were showed in table 6.

**Table 6:** Differences of calculated heads of bare slope vs. observed values of grass land/straw mulching.

Scenario	Average change(cm)	CM_max_/cm	RM/cm	ARM/cm	RMS/cm	NRMS/%
Grass Land	3.91	−11.8	−3.6	4.1	4.5	14.1
Straw Mulching	7.63	−20.5	−7.3	7.4	7.8	23.4

**Figure 9:**
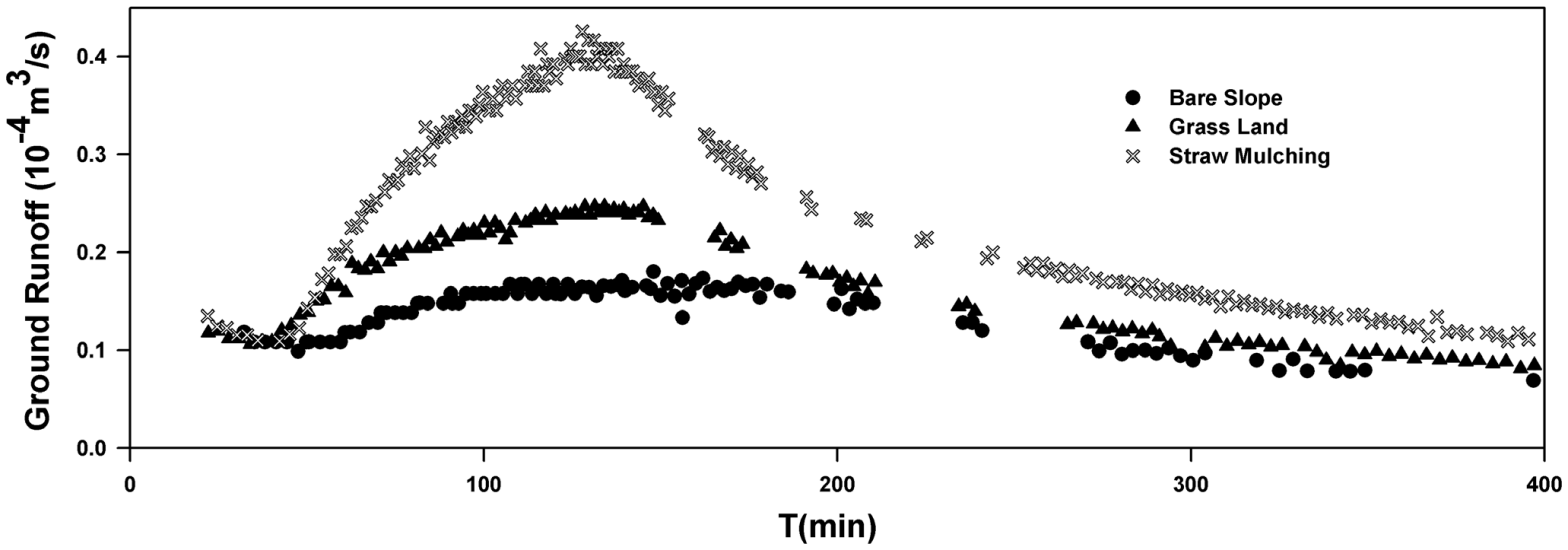
The groundwater runoff of bare slope vs. the discharge of lawn and straw mulching.

It was explicit from the curve of figure 9 that the soil and water conservations had great influence upon the groundwater runoff. Straw mulching showed the higher runoff, followed by grassland. It was illustrated by further analysis that average flow was 0.14×10^−4^ m^3^/s in the bare slope scenario, 0.17×10^−4^ m^3^/s in the lawn scenario, and 0.26×10^−4^ m^3^/s in the straw mulching scenario, during the simulation period. So the average runoff increased by 1.23 and 1.87 times through measures of lawn and straw mulching.

In summary, both grass and straw mulching scenarios played an important role in reducing the surface runoff and increasing the underground runoff. The effects of measures on redistribution of rainfall runoff was mainly due to the rainfall infiltration rata was changed. The grass and straw mulching dispersed the large raindrops into small raindrops, which reduced the actual rainfall intensity on the ground [38]–[42] and made more precipitation meet the condition of infiltration. But different soil and water conservation measures had different effects. In this study, straw mulching has more significant hydrological effects than lawn. The main difference between the two scenarios was that the straw mulching scenario had less bare land area compared to lawn scenario.

## Conclusions

Based on the principle of water balance, a linked approach for SCS model and Visual MODFLOW was conducted to assess the impact of soil and water conservation on the surface water and groundwater runoff. The calibration results showed that the predicted results matched well with the observed data. Therefore, three land use management scenarios were simulated on the sand-box model to assess the effect of bare slope converted to grass land and straw mulching on water volume, hydraulic head, runoff process of groundwater and surface water.

Soil and water conservation measures could reduce surface runoff effectively. Under the 120 mm rainfall, 60 mm/h rainfall intensity, 5 m^2^ area, 3° slope conditions, the comparative results indicated that decrease in surface runoff and increase in subsurface runoff coincided with the land-use converted from bare slope to grass land and straw mulching. Compared to the bare slope, the benefits of surface water reduction by these two measures were 57.8% and 92.4% correspondingly. Not only the individual soil and water conservation measure but also the comprehensive management of the small watershed had a significant benefit of surface water reduction. The comparative results of 12 groups (24) of controlled and uncontrolled small watersheds showed that the benefits of surface water reduction varied from 14.71% to 88.19% while the corresponding average value was 44.09%.

Soil and water conservation measures could promote rainfall recharge groundwater. Under the same condition, compared to the bare slope, the amount and the benefit of groundwater recharge increased by 0.064 m^3^ and 22.7% respectively in the lawn scenario. Accordingly, they were 0.209 m^3^ and 60.4% in the straw mulching scenario. For the runoff, the average flow of straw mulching was highest, the grassland took second place, the bare slope was the lowest, and the average runoff increased by 1.23 and 1.87 times through measures of lawn and straw mulching.

It was concluded that the soil and water conservation played an important role in weakening the surface runoff and strengthening the underground runoff. Meanwhile the groundwater flow model coupled with the surface water model used in this study, when properly validated, could be used as a tool in the evaluation of soil and water conservation measures on surface water and groundwater resources, and this approach could provide a thought for the study in a watershed scale to help decision-makers manage groundwater resources.
